# Structured Symptom Assessment in Dermato-Oncology Patients—A Prospective Observational Study of the Usability of a Symptom Questionnaire

**DOI:** 10.3390/cancers17233763

**Published:** 2025-11-25

**Authors:** Martin Gschnell, Jannis Thole, Lisa Kroenig, Marianne Arz, Armin Bender, Michael Hertl, Christian Volberg

**Affiliations:** 1 Department of Dermatology and Allergology, Marburg University Hospital, Philipps University of Marburg, 35043 Marburg, Germany; gschnell@med.uni-marburg.de (M.G.);; 2 Department of Anaesthesiology, Intensive Care and Emergency Medicine, German Armed Forces Hospital Westerstede, 26655 Westerstede, Germany; 3 Department of Anaesthesiology & Intensive Care Medicine, Faculty of Medicine, Philipps University of Marburg, 35043 Marburg, Germany

**Keywords:** symptom management, dermato-oncology, symptom questionnaire, palliative care, living will

## Abstract

Background: A focused anamnesis of symptoms is essential in medical practice, especially in oncology, where new or increasing symptoms often indicate disease progression or therapy side effects. However, there are only a few studies in dermato-oncology that address the question of how symptoms should ideally be recorded. There are no validated questionnaires specifically tailored to the needs of dermato-oncology. Patients and Methods: Over a one-year period, all patients at the skin cancer centre were given a standardised questionnaire to record relevant symptoms before consultation. This was followed by the attending dermato-oncologist completing an additional section regarding therapeutic measures based on the reported symptoms. Results: 809 patients with 1879 consultations were included. In most patient contacts, patients did not report an increase in symptoms (n = 840; 44.7%). Fatigue, restlessness/anxiety, pruritus, and pain were the most recorded distressing symptoms. The chi-square test revealed that increased symptoms led to significantly more frequent interventions. Overall, symptom relief was observed at follow-up. Analysis using Generalised Estimating Equations showed patients benefited significantly from interventions for sleep disorders. Subgroup analysis showed that patients in ongoing therapy benefitted significantly from interventions for pain and nausea. Those in advanced disease stages benefitted significantly from interventions for pain. In those groups, if symptoms were explicitly stated as having worsened using the questionnaire, measures taken by physicians also showed significant benefit for obstipation and nausea along with pruritus. Conclusions: Targeted symptom screening is feasible and provides valuable insights for guiding diagnostic or therapeutic interventions. However, the questionnaire is most useful at higher tumour stages, as it is only effective when distressing symptoms are present.

## Simple Summary

Skin cancer patients often experience symptoms that can signal changes in their disease or side effects from treatment, but doctors currently lack a simple, reliable way to record these symptoms early and consistently. This study set out to test whether a standardised symptom checklist, completed by patients before seeing their doctor, could help identify troubling symptoms more effectively and guide treatment decisions. By using this tool over a full year in a specialised skin cancer centre, the researchers aimed to understand which symptoms are most common, whether reporting worsening symptoms leads to meaningful medical interventions, and how much patients ultimately benefit from these measures. The findings may help the research community develop better symptom-monitoring tools in dermato-oncology and improve patient care by ensuring that symptoms are recognised and managed earlier and more consistently.

## 1. Introduction

A targeted medical history can correctly identify over 80% of existing diseases [1,2,3]. Knowledge of symptoms is therefore essential for the treating physician to provide targeted therapy [4]. On the one hand, this is the only way to achieve adequate symptom control; on the other hand, an increase in symptoms can also indicate disease progression. In oncological diseases in particular, new symptoms can indicate tumour growth or the occurrence of new metastases, as well as impaired organ function due to the tumour or metastases [5]. Especially in advanced palliative disease, almost all oncological patients suffer from one or more distressing symptoms, most commonly pain, confusion, anorexia or fatigue [6]. Dermato-oncology patients with advanced tumour disease usually suffer from fatigue, sleep disorders and anxiety. More than 60% of patients describe these symptoms as distressing [7]. Specific side effects of immune checkpoint inhibitors occur in 86–96% of patients undergoing treatment. These can manifest in any organ system but are most commonly observed in the skin, intestines, liver and endocrine organs. Severe to life-threatening side effects occur in up to 59% of patients receiving approved combination therapies, which makes screening patients for side effects or symptoms that may indicate them absolutely essential. Early intervention can hereby prevent serious complications [8,9].

However, symptoms are not always reported to the treating physician, with many reasons for this:

- In some cases, patients may be unaware that symptoms can indicate disease progression or be a relevant side effect of therapy.
- They may be afraid to report symptoms because they fear the consequences, such as disease progression and subsequent termination of therapy, or approaching the end of life.
- They feel uncomfortable discussing intimate problems such as diarrhoea or loss of libido.
- They do not recognise a symptom as such (e.g., fatigue or sleep disorders).
- They are too agitated during the consultation with the treating physician and forget to mention their problems [10,11,12,13,14].

In everyday clinical practice, there are two main approaches to assessing symptoms: the medical history interview and the use of appropriate questionnaires. These are often used in combination. Both approaches have their advantages and disadvantages. During a medical consultation, targeted questions can generate more detailed information about the nature and severity of symptoms. A predefined questionnaire cannot allow for this to the same extent because of the answer options that have been provided and the limited space for free text responses. Conversely, patients can complete a questionnaire about their symptoms at their own pace, so they will not risk forgetting to mention anything to their doctor amid the hustle and bustle of everyday life or the excitement of a personal consultation. Additionally, questionnaires allow for a more neutral approach to sensitive topics than personal consultations. Progress can be documented if patients are seen regularly and the questionnaires are filed in their records. However, patients must be literate and have a command of the language in order to complete a questionnaire [15,16].

As no survey has yet been conducted on the usefulness of symptom questionnaires and structured symptom assessments in dermato-oncology, the initial aim of this feasibility study was to determine if the questionnaire could be used during consultations and if its usefulness differed when surveying different tumour types and stages. The additional research question being investigated is whether action taken by doctors follows symptoms reported by patients and whether this results in symptom relief at the follow-up appointment.

## 2. Materials and Methods

This was a single-centre, prospective, observational study conducted at the Skin Cancer Centre of the Department of Dermatology and Allergology at Marburg University Hospital. It was approved by the Ethics Committee of Philipps University of Marburg (31 May 2022, file number 65/22) and registered in the German Clinical Trials Register on 6 June 2022 (DRKS00029196). The study was conducted between 1 June 2022 and 31 May 2023.

The inclusion criteria were:

- Treatment at the Skin Cancer Centre
- Confirmed diagnosis of a malignant dermatological tumour
- Sufficient knowledge of German and the ability to read and write.

Exclusion criteria were:

- Age ≤ 10 years
- Impaired cognition
- Insufficient knowledge of German
- Incomplete questionnaire.

### 2.1. Development of the Questionnaire

Following a preparatory phase involving a review of international literature on the subject, a team of dermato-oncologists, dermatologists and palliative care specialists developed the questionnaire. The main focus during the development of the questionnaire was its clinical usefulness to the team at the Skin Cancer Centre.

The questionnaire consisted of two sections: one related to symptoms and the other to treatment. Patients were asked to rate the severity of various relevant symptoms (e.g., pain, dyspnoea, nausea, diarrhoea, constipation, fatigue, itchiness, sleep problems, depression/anxiety/inner restlessness) on 11-point Likert scales (Numeric Rating Scale (NRS); 0 = no symptom, 10 = strongest conceivable symptom severity) and to indicate whether the symptom had worsened since their last visit to the skin cancer centre using a decision question (yes/no). When assessing the increase or decrease in symptom severity using an 11-point NRS, a difference of more than two points from the previous value can be considered relevant [17]. Additionally, questions about unintentional weight loss and night sweats were asked with dichotomous response options. Finally, patients were asked if they had written a living will at the time of their appointment or if they would like information on how to do so.

The symptom-related section of the questionnaire was based on the Edmonton Symptom Assessment Scale (ESAS), which is a frequently used questionnaire in palliative care. It is used to assess nine predominant symptoms, including pain, fatigue, nausea, depression, anxiety, drowsiness, appetite, general well-being, and dyspnoea, using an 11-point Likert scale. The ESAS was developed in 1991 and has since been psychometrically validated and translated into more than 20 languages, including German [18,19,20]. However, the present study focused on the aforementioned symptoms, which occur particularly frequently in dermato-oncological patients or may indicate side effects of immune or targeted therapies [7,9].

The treating dermato-oncologist completed the treatment-specific part of the questionnaire, which included information on tumour stage and type of skin cancer, any ongoing drug or interventional cancer-specific therapy being administered, and whether a living will was on file at the Skin Cancer Centre. It was also recorded whether measures had been taken to address the patient’s reported symptoms and, if so, what these measures were.

The English translation of the questionnaire can be found in the Supplementary Material.

### 2.2. Data Collection

As part of a new standard procedure in clinical routine, the questionnaires were handed out to every patient at the Skin Cancer Centre upon registration and could be completed during the waiting period. Patients with multiple appointments were surveyed more frequently, with data collected each time to record symptom progression. This was a prospective observational study, and the questionnaire was used as part of routine clinical treatment and not as part of a study, so no additional information or consent from patients was required for data processing, since the data was processed anonymously. This approach also aligns with German legislation, which states that anonymised patient data from routine clinical care does not require the patient’s additional consent.

### 2.3. Data Analysis

Data analysis was performed using IBM^®^ SPSS^®^ Statistics software, version 29.0. The chi-square test was used to demonstrate correlations between dichotomous variables. The Shapiro–Wilk test was used to assess the normality of quantitative data. To account for repeated measurements of symptom-specific relief, Generalised Estimating Equation (GEE) were employed, which consider intra-individual correlation of patient contacts. The dependent variable was metrically called symptom relief, and predictors included whether measures were taken. This was analysed for the whole study population as well as specifically for those who reported exacerbations. In addition, subgroup analyses were conducted for contacts of patients undergoing interventional or drug tumour therapy and contacts of patients in advanced disease stages (AJCC-TNM classification 2017 stages III + IV). The significance level was set at α = 0.05. Microsoft^®^ Excel version 2406 was used to create graphs. In line with the recommendations of the EQUATOR Network (Enhancing the Quality and Transparency of Health Research), the STROBE (Strengthening the Reporting of Observational Studies in Epidemiology) checklist was used to prepare the manuscript [21].

## 3. Results

### 3.1. Patient Characteristics

**Figure 1:**
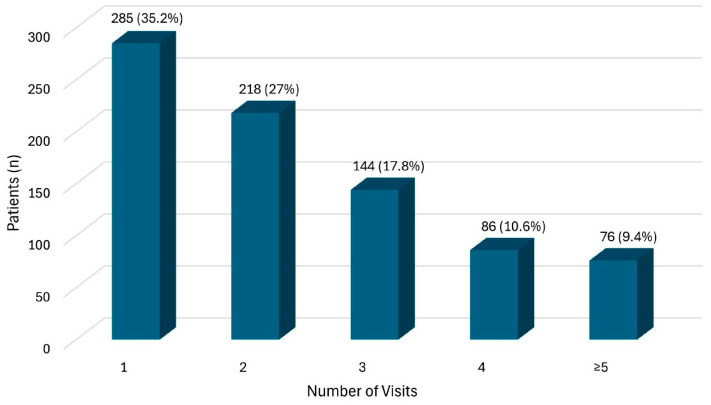
Number of patient contacts per patient during the survey period (n = 809).

This one-year prospective observational study included a total of 809 patients, while 14 individuals could not be included. A total of 1879 patient contacts were recorded and evaluated. Figure 1 shows how often patients attended appointments. The median age of the entire sample was 68 years (Q1: 57 yrs; Q3: 79 yrs; R = 86 years; min: 11 years; max: 97 years) and 56.5% of participants (n = 457) were male. Further demographic and disease-related data on the study participants at the time of their inclusion in the study can be found in Table 1 and Table 2. The tables represent data derived from the first patient encounter during our study period.

### 3.2. Descriptive Analysis

**Figure 2:**
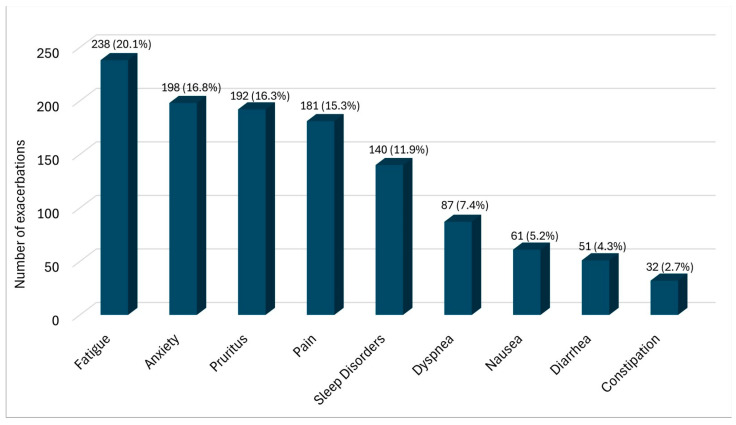
Patients’ symptoms described as increasing. An increase was defined as a difference ≥2 points on the NRS compared to the previous value. (n = 1180 contacts).

In most patient contacts, patients did not report an increase in symptoms (n = 840; 44.7%). In 165 cases (8.8%), one symptom was described as having worsened, and in 87 cases (4.6%), two symptoms were described as having worsened. Patients reported a worsening of three or more symptoms in 109 cases (5.8%). In 678 cases (36%), no information was provided. In total, symptoms were described as increasing 1180 times compared to the previous appointment. Of these, 841 subjective exacerbations occurred in patient contacts in which three or more symptoms were described. These exacerbations primarily occurred in relation to fatigue (n = 238), restlessness/anxiety (n = 198), pruritus (n = 192), and pain (n = 181). Figure 2 provides an overview of the symptoms primarily described as increasing.

**Figure 3:**
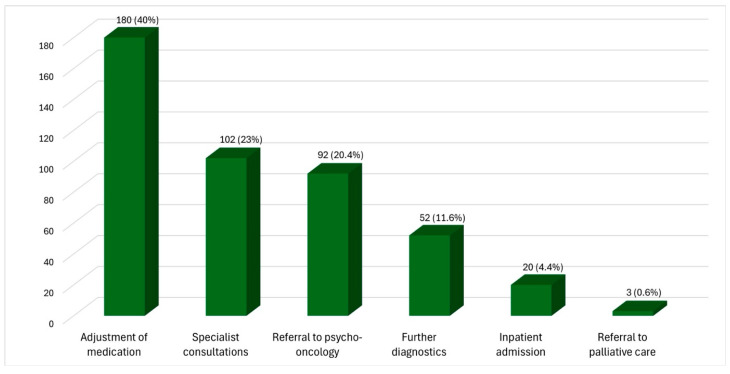
Measures initiated based on the symptoms described in the questionnaire (n = 373).

Dermato-oncologists initiated measures in 373 patient contacts. In most cases (n = 308), only one measure was taken; in 54 cases, two measures were taken; and in 10 cases, three or more measures were taken. Figure 3 provides an overview of these measures. The most common measure by far was adjusting symptom-related medication (n = 180), followed by consultations or referrals to specialists (n = 102) and referrals to psycho-oncological care services (n = 92). Inpatient admissions (n = 20) and referrals to palliative care services (n = 3) were extremely rare.

Of the 809 patients, 381 (47.1%) had a living will at their first appointment, while 113 requested information material on this subject. During the observation period, 27 patients reported that they had subsequently drawn up a living will.

### 3.3. Response to Reported Increase in Symptoms

Global analysis showed that patients who reported one or more symptoms as being worse than at the previous appointment were significantly more likely to receive treatment (Pearson-Chi^2^: 118.23, *p* < 0.001, Phi = 0.382). This correlation was equally significant when the analysis was restricted to patients receiving tumour-specific therapy (Pearson-Chi^2^: 20.87, *p* < 0.001, Phi = 0.372). The same applies to the subgroup of patients with advanced tumour stages III or IV according to the AJCC staging system (Pearson-Chi^2^ = 58.7, *p* < 0.001, Phi = 0.452).

When the individual symptoms are considered, differences emerge in the measures taken in response to symptom increases. For instance, patients reporting an exacerbation of pain were significantly more likely to receive all recorded measures (*p* < 0.001; Phi = 0.23; connection to palliative care: *p* = 0.002), except for connection to psycho-oncological care (*p* = 0.266), than those without an increase in pain. By contrast, psycho-oncological care was initiated significantly more frequently in patients who reported increasing sleep disturbances, low mood, inner restlessness or anxiety than in those without these symptoms, though the effect size remains weak (*p* < 0.001, Phi = 0.108). This is more evident in the adjustment of symptom-related medication in response to a reported increase in pruritus: not only is the correlation significant compared to the group without an increase in pruritus, but the effect size is also robust (*p* < 0.001; Phi = 0.316).

### 3.4. Effect of Measures Taken

For patients who reported an increase in one or more symptoms since their last appointment at the Skin Cancer Centre, the extent to which they experienced relief was examined using the NRS at the follow-up appointment. All variables significantly deviated from a normal distribution (*p* < 0.001), largely due to the extreme centralisation of values around the median, resulting in highly skewed distributions (see Table 3). This was true for all patients reporting an increase in one or more symptoms, as well as analysed sub-groups of patients actively undergoing drug or interventional tumour therapy. Consequently, non-parametric tests were used.

Across the full study cohort, regardless of whether specific symptoms were marked as increasing in the questionnaire, taking measures did not have a significant impact across the assessed symptom range with one exception: the relief of sleep disorders. The GEE analysis revealed a significant effect of taken measures (Wald-Chi^2^ = 4.39, *p* = 0.036). Patient contacts without intervention showed a slight increase in mean symptom severity of 0.05, while patients with measures taken showed a mean relief of −0.24.

Similar observations were made when only the relief of symptoms following interventions was analysed, restricted to symptoms specifically reported as increasing, though the mean relief was higher. Only the relief of sleep disorders was statistically significant (Wald-Chi^2^ = 4.56, *p* = 0.033); mean relief for patients without measures was −1.09, while mean relief for patients after intervention was −2.68.

Subgroup analysis for patients undergoing interventional or drug-based tumour therapy revealed significant effects of measures taken on pain relief (Wald-Chi^2^ = 12.91, *p* < 0.001) and nausea relief (Wald-Chi^2^ = 5.01, *p* = 0.025). Patients with nausea and no measures taken by the physician showed a mean symptom increase of 0.14, while patients with measures taken showed a relief of −0.34. Respectively, patients with pain and no measures taken showed a mean increase in pain severity of 0.31, while those with interventions showed a decrease of −0.7. This was the case whether symptoms were reported to have exacerbated or not. Interestingly, in patients who explicitly stated the analysed symptom as having worsened since the last consultation, another symptom became statistically significant: relief of obstipation (Wald-Chi^2^ = 11.7, *p* < 0.001). Considering the mean values after measures were taken, those did not correspond to a relief per se but an attenuation of symptom increase; the mean increase without measures was 5.4, while the mean increase with measures was 3.3.

Patients in advanced tumour stages (defined as AJCC TNM classification [2017] stadiums III and IV) also significantly benefited from measures taken regarding pain relief (Wald-Chi^2^ = 5.54, *p* = 0.019). Mean pain relief after measures taken was −0.4, while those without intervention showed a slight mean increase in pain of 0.06. However, if certain symptoms were explicitly stated as having worsened in the questionnaire, effect measures taken in this subgroup became significant in two more dimensions: relief of nausea (Wald-Chi^2^ = 4.93, *p* = 0.026) and relief of pruritus (Wald-Chi^2^ = 3.93, *p* = 0.047). The mean relief of symptoms of measures taken was −0.38 for nausea and −0.5 for pruritus, respectively.

## 4. Discussion

Accurately and quickly recording symptoms can sometimes be difficult in everyday clinical practice. As outlined in the introduction, various barriers can hinder symptom assessment, affecting both patients and practitioners. Our data show that a symptom questionnaire can be a useful tool in dermato-oncology, facilitating the targeted recording of symptoms and the initiation of appropriate treatment. However, the analysis also shows that the questionnaire is more useful for patients with advanced cancer, as those with earlier stages experience significantly fewer distressing symptoms. In a Germany-wide survey of certified Skin Cancer Centres and dermatological oncology practices conducted by our study group, 97.8% of participating institutions stated that they regularly screen patients for distressing symptoms. This is performed in 54.6% of cases during the medical history, in 40.9% of cases in combination with targeted questionnaires, and in just 4.6% of cases based solely on questionnaires [22].

Higher tumour stages, metastasis and tumour-specific therapy are particularly prevalent among patients with the palliative stage of illness. For these patients, tumour disease often results in a reduced quality of life and the need for effective symptom management. Studies on other tumour types have shown that early palliative care can improve quality of life and prolong survival [23,24,25]. Palliative care should not be viewed as end-of-life treatment; rather, it should be incorporated into the treatment process for patients with incurable tumour disease from the outset. It is therefore important to identify those who require appropriate treatment or have other end-of-life needs (e.g., setting up a living will or accessing psycho-oncological care).

In palliative medicine, there are already validated symptom questionnaires. Examples include the Edmonton Symptom Assessment Scale (ESAS) and the Minimal Documentation System (MIDOS), the latter of which is widely used in German-speaking countries. The ESAS questionnaire, which served as a template for the questionnaire used in this study, uses an 11-point Likert scale to assess symptoms, while the MIDOS questionnaire uses a four-point categorical scale to facilitate processing for palliative care patients [18,20]. As described in the methodology, our questionnaire for assessing symptoms in dermato-oncology patients combines and extends various existing questionnaires to also assess dermatologically relevant and therapy-specific problems [7,9]. In addition, patients are asked to self-assess whether their symptoms have worsened since their last treatment appointment to illustrate disease progression. When assessing the increase or decrease in symptom severity using an 11-point NRS, a difference of more than two points from the previous value can be considered relevant [17]. It is therefore also interesting to know how patients subjectively perceive their symptoms. Evaluation of the collected data shows that it is this self-assessment by patients in particular that has led to a significantly more frequent initiation of appropriate treatments.

The second part of the questionnaire, completed by the attending physician after the consultation with the patient, enables the measures initiated for follow-up appointments to be recorded, thus ensuring adequate documentation of the course of treatment.

In addition to assessing distressing symptoms, the requirements catalogue for the certification of German Skin Cancer Centres includes screening for psychosocial needs [22,26]. In our cohort, a significantly higher uptake of psycho-oncological counselling was observed among those who responded positively to questions about an increase in sleep disorders, low mood, inner restlessness or anxiety. This is of particular interest as psychosocial stress is often overlooked by both practitioners and patients. However, psychosocial stress can result in a reduced quality of life for those affected and, in the worst case, may lead to the development of depression or anxiety disorders [27]. The so-called ‘Distress Thermometer’ is a valid instrument for objectively assessing the psychosocial stress experienced by patients. It is an 11-point Likert scale in the form of a thermometer, and a score of 4 or above indicates that psychological or psycho-oncological counselling should be initiated [28,29]. In this context, discussions about care planning in the event of a deterioration in health are also helpful and are often requested by patients. Unfortunately, these discussions are rarely conducted by treating oncologists in practice [7,30]. However, a lack of discussion and knowledge about the disease’s status can result in poorer care, as patients are unable to address relevant end-of-life issues and take appropriate precautions, e.g., drawing up a living will [31]. This often results in unwanted overtreatment at the end of life, placing a burden on close relatives who have to make decisions without knowing the patient’s presumed wishes [32]. Therefore, it is helpful if the staff of the Skin Cancer Centre or the treating dermato-oncologist has relevant information about advance directives and can offer assistance in drawing up living wills or Advance Care Planning (ACP). ACP has proven to be a helpful tool for carrying out meaningful care planning with patients, as well as for involving relatives in the discussion process, thereby breaking down barriers to communication [33]. ACP can be carried out with appropriately trained counsellors or integrated into psycho-oncological care [34]. Appropriate offers should be made to patients and relatives on an ongoing basis with low thresholds. Our questionnaire’s question about whether the patient has drawn up a living will or would like information on the subject can be understood as such a subtle message.

## 5. Limitations

The questionnaire does not differentiate between symptoms reported by patients that are tumour-specific and those unrelated to the dermato-oncological disease. For example, it is not possible to distinguish between dyspnoea in patients due to COPD or due to lung metastases. Although the items in the questionnaire are taken from validated sources, the questionnaire itself has not been psychometrically validated, meaning that the results can only be interpreted with caution. Furthermore, this is a single-centre study, meaning that the results cannot be applied to other clinics or countries. The decision to combine the six individual measures into one dichotomous variable of ‘measures taken/no measures taken’ (see Figure 3) was made because, despite a large number of patient contacts being analysed, the sample sizes for testing each symptom against each measure were too small. This makes it difficult to specifically attribute effects to one of the described measures. There is no comparison group that was treated under standard conditions and can be used as a reference. To make a statement about usability in different groups (e.g., patients with malignant melanoma or basal cell carcinoma), a larger sample size is required for corresponding subgroup analyses. Follow-up studies are therefore necessary to test hypotheses.

## 6. Conclusions 

The data from this study demonstrate that systematically recording symptoms as part of dermato-oncological treatment using a symptom questionnaire is straightforward and practical and can inform targeted therapeutic decisions. However, the data also show that the questionnaire is not suitable for all patients, as it only provides practitioners with helpful information in advanced tumour stages or when symptoms are present. Further studies should be conducted to determine the most appropriate groups and circumstances for using the questionnaire. The questionnaire should also be adapted to the necessary circumstances and validated for the type of survey. Furthermore, staff must be willing to incorporate patient-provided data into treatment discussions and initiate appropriate measures. Implementing these points and establishing a questionnaire as part of routine care would enable physicians to recognise distressing symptoms more quickly for the benefit of patients.

## Figures and Tables

**Table 1:** Demographic and disease-related data (n = 809).

	Male	Female
	n = 457 (56.5%)	n = 352 (43.5%)
Age (Mdn; R; Q1/Q3) *	71; 82 (11–93); 61/71 years	63; 85 (12–97); 53/75 years
**Diagnose**		
Malignant melanoma	272 (59.5%)	247 (70.2%)
Squamous cell carcinoma	76 (16.6%)	24 (6.8%)
Basal cell carcinoma	16 (3.5%)	8 (2.3%)
Cutaneous sarcomas	30 (6.6%)	23 (6.5%)
Merkel cell carcinoma	12 (2.6%)	12 (3.4%)
Cutaneous lymphomas	37 (8.1%)	27 (7.7%)
Other	14 (3.1%)	11 (3.1%)
**Ongoing tumour-specific therapy**		
None	399 (87.3%)	321 (91.2%)
Palliative	40 (8.8%)	21 (6.0%)
Adjuvant	18 (3.9%)	10 (2.8%)

* Mdn = Median; R = Range; Q1/Q3 = Quartile1/Quartile3.

**Table 2:** Clinical stage according to the 8th edition of the AJCC TNM classification (2017) (n = 809).

Clinical Stage According to TNM Classification	
**Malignant melanoma**	
I	185 (22.9%)
II	126 (15.9%)
III	138 (17.1%)
IV	70 (8.7%)
n.a.	0
**Squamous cell carcinoma**	
I	36 (4.5%)
II	36 (4.5%)
III	17 (2.1%)
IV	11 (1.4%)
n.a.	0
**Basal cell carcinoma**	
n.a. *	24 (3.0%)
**Cutaneous sarcomas**	
I	11 (1.4%)
II	0
III	0
IV	0
n.a.	42 (5.2%)
**Merkel cell carcinoma**	
I	9 (1.1%)
II	4 (0.5%)
III	9 (1.1%)
IV	2 (0.2%)
n.a.	0
**Cutaneous T-cell lymphomas**	
I	26 (3.2%)
II	5 (0.6%)
III	6 (0.7%)
IV	4 (0.5%)
n.a.	2 (0.2%)
**Cutaneous B-cell lymphomas**	
I	9 (1.1%)
II	0
III	0
IV	0
n.a.	12 (1.5%)
**Other neoplasms**	
I	2 (0.2%)
II	0
III	3 (0.4%)
IV	7 (0.9%)
n.a.	13 (1.6%)

* The AJCC TNM criteria are not clinically relevant for predicting the outcome of basal cell carcinoma, which is why the stage is not recorded. n.a. = not applicable.

**Table 3:** Symptom relief calculated as difference between reported symptom severity by 11-point Likert Scale between visits. Calculation based upon 936 analysed patient contacts.

	Pain Relief	Dyspnoea Relief	Nausea Relief	Diarrhoea Relief	Obstipation Relief	Fatigue Relief	Pruritus Relief	Relief of Sleep Disorders	Relief of Anxiety
**5. percentile**	−3	−2	−1	−2	−2	−3	−3	−3	−3
**10. percentile**	−2	−1	0	−0.3	0	−2	−2	−2	−2
**25. percentile**	0	0	0	0	0	−1	0	0	0
**Median**	0	0	0	0	0	0	0	0	0
**75. percentile**	0	0	0	0	0	1	0	0	0
**90. percentile**	2	1	0	1	0	2	2	2	2
**95. percentile**	3	3	1	2	1	3	3	3	3
**Range**	20	17	16	19	18	18	20	18	18

## Data Availability

The data are available upon reasonable request from the authors.

## Supplementary Materials

The following supporting information can be downloaded at: https://www.mdpi.com/article/10.3390/cancers17233763/s1, English translation of the questionnaire used.
