# Supplementary material for: Structured Symptom Assessment in Dermato-Oncology Patients—A Prospective Observational Study of the Usability of a Symptom Questionnaire
Source: Cancers (Basel). 2025 Nov 25;17(23):3763. doi: 10.3390/cancers17233763 (PMC12691204; doi:10.3390/cancers17233763)
Supplement: Supplementary file 1 [file cancers-17-03763-s001.zip › cancers-3942108-supplementary.pdf]

Date: _ _ . _ _ . _ _ _ _

Dear patient,

You have an appointment with your dermato-oncologist today. To ensure that all aspects of your condition can be treated in the best possible way and to simplify the discussion with your doctor, we kindly ask you to complete this questionnaire while you are waiting.

Thank you very much,
Your team at the Marburg Skin Cancer Center

1. **How severe was your pain during the past 2 weeks?**

[0]	[1]	[2]	[3]	[4]	[5]	[6]	[7]	[8]	[9]	[10]
No Pain										Worst imanginable pain

Has the pain increased since your last visit with us?

Yes O No O

If you had pain, where was it located?

____

2. **How severe was your shortness of breath during the past 2 weeks?**

[0]	[1]	[2]	[3]	[4]	[5]	[6]	[7]	[8]	[9]	[10]
No shortness of breath										Worst imaginable shortness of breath

Has this shortness of breath increased since your last visit with us?

Yes O No O

3. How severe was your nausea during the past 2 weeks?

[0]	[1]	[2]	[3]	[4]	[5]	[6]	[7]	[8]	[9]	[10]
No nausea										Worst imaginable nausea

Has this nausea increased since your last visit with us?

Yes O No O

4. How severe was your diarrhea during the past 2 weeks?

[0]	[1]	[2]	[3]	[4]	[5]	[6]	[7]	[8]	[9]	[10]
No diarrhea										Worst imaginable diarrhea

Has this diarrhea increased since your last visit with us?

Yes O No O

5. How severe was your constipation during the past 2 weeks?

[0]	[1]	[2]	[3]	[4]	[5]	[6]	[7]	[8]	[9]	[10]
No constipation										Worst imaginable constipation

Has this constipation increased since your last visit with us?

Yes O No O

6. How severe was your daytime fatigue during the past 2 weeks?

[0]	[1]	[2]	[3]	[4]	[5]	[6]	[7]	[8]	[9]	[10]
No fatigue										Worst imaginable Fatigue

Has this daytime fatigue increased since your last visit with us?

Yes O No O

7. How severe was your itching during the past 2 weeks?

[0]	[1]	[2]	[3]	[4]	[5]	[6]	[7]	[8]	[9]	[10]
No itching										Worst imaginable itching

Has this itching increased since your last visit with us?

Yes O No O

8. **How severe were your sleep problems during the past 2 weeks?**

[0]	[1]	[2]	[3]	[4]	[5]	[6]	[7]	[8]	[9]	[10]
No sleep problems										Worst imaginable sleep problems

Have these sleep problems increased since your last visit with us?

Yes O No O

9. **How severe were feelings of sadness, anxiety, or inner restlessness during the past 2 weeks?**

[0]	[1]	[2]	[3]	[4]	[5]	[6]	[7]	[8]	[9]	[10]
No feelings of sadness, anxiety or inner restlessness										Worst imaginable feelings of sadness, anxiety or inner restlessness

Have these feelings increased since your last visit with us?

Yes O No O

10. **Have you unintentionally lost weight since your last visit with us?**

Yes O No O If yes, how much?: __________

11. **Do you suffer from severe night sweats?**

Yes O No O

12. **Do you have an advance healthcare directive?**

Yes O No O

If no, would you like information on how to create one?

Yes O No O

_ _

_ _

To be completed by the treating physician::

1. **Tumor entity:**

O Malignant Melanoma O Squamous cell carcinoma O Basal cell carcinoma

O B-cell lymphoma O T-cell lymphoma O Cutaneous sarcoma

O Other________________________

2. **Clinical tumor stage (AJCC 2017):**

O Not applicable O Stage I O Stage II O Stage III O Stage IV

3. **Current tumor therapy:**

O None O Adjuvant O Palliative

O Ipilimumab/Nivolumab O Nivolumab O Pembrolizumab

O Cemiplimab O Avelumab O Mogamulizumab

O Encorfenib/Binemitinib O Vemurafenib/Cobimetinib O Dabrafenib/Trametinib

O Vismodegib O Sonedigib

O T-VEC O Tebentafusp O Other: __________

4. **Is the patients advance directive on file at the Skin Cancer Centre?**

O Yes O No

5. **Measures taken based on symptoms?**

O Yes O No

If yes, which ones?:

O Medication for symptom control adjusted ____

O Diagnostics ordered: O CT O Ultrasound O MRI O Other: _______

O Psycho-oncology referral

O Information material provided for an advance directive

O Inpatient admission

O Specialist referral: _____________

O Palliative care referral
